# Transcriptome-wide analyses indicate mitochondrial responses to particulate air pollution exposure

**DOI:** 10.1186/s12940-017-0292-7

**Published:** 2017-08-18

**Authors:** Ellen Winckelmans, Tim S Nawrot, Maria Tsamou, Elly Den Hond, Willy Baeyens, Jos Kleinjans, Wouter Lefebvre, Nicolas Van Larebeke, Martien Peusens, Michelle Plusquin, Hans Reynders, Greet Schoeters, Charlotte Vanpoucke, Theo M de Kok, Karen Vrijens

**Affiliations:** 1 Centre for Environmental Sciences, Hasselt University, Agoralaan gebouw D, B-3590 Diepenbeek, Belgium; 2 Department of Public Health & Primary Care, Leuven University, Leuven, Belgium; 3 Provincial Institute for Hygiene, Antwerp, Belgium; 4 Department of Analytical and Environmental Chemistry, Vrije Universiteit Brussel, Brussels, Belgium; 5 Department of Toxicogenomics, Maastricht University, Maastricht, The Netherlands; 6 Flemish Institute for Technological Research, Mol, Belgium; 7 Department of Radiotherapy and Nuclear Medicine, Ghent University, Ghent, Belgium; 8 Environment, Nature and Energy Department, Flemish Government, Brussels, Belgium; 9 Department of Biomedical Sciences, University of Antwerp, Antwerp, Belgium; 10 Institute of Public Health, Department of Environmental Medicine, University of Southern Denmark, Odense, Denmark; 11 Belgian Interregional Environment Agency (IRCEL), Brussels, Belgium

**Keywords:** Ambient air pollution, Particulate matter, Transcriptome-wide analyses, Sex-specific, mitochondria

## Abstract

**Background:**

Due to their lack of repair capacity mitochondria are critical targets for environmental toxicants. We studied genes and pathways reflecting mitochondrial responses to short- and medium-term PM_10_ exposure.

**Methods:**

Whole genome gene expression was measured in peripheral blood of 98 adults (49% women). We performed linear regression analyses stratified by sex and adjusted for individual and temporal characteristics to investigate alterations in gene expression induced by short-term (week before blood sampling) and medium-term (month before blood sampling) PM_10_ exposure. Overrepresentation analyses (ConsensusPathDB) were performed to identify enriched mitochondrial associated pathways and gene ontology sets. Thirteen Human MitoCarta genes were measured by means of quantitative real-time polymerase chain reaction (qPCR) along with mitochondrial DNA (mtDNA) content in an independent validation cohort (*n* = 169, 55.6% women).

**Results:**

Overrepresentation analyses revealed significant pathways (*p*-value <0.05) related to mitochondrial genome maintenance and apoptosis for short-term exposure and to the electron transport chain (ETC) for medium-term exposure in women. For men, medium-term PM_10_ exposure was associated with the Tri Carbonic Acid cycle. In an independent study population, we validated several ETC genes, including *UQCRH* and *COX7C* (*q*-value <0.05), and some genes crucial for the maintenance of the mitochondrial genome, including *LONP1* (*q*-value: 0.07) and *POLG* (*q*-value: 0.04) in women.

**Conclusions:**

In this exploratory study, we identified mitochondrial genes and pathways associated with particulate air pollution indicating upregulation of energy producing pathways as a potential mechanism to compensate for PM-induced mitochondrial damage.

**Electronic supplementary material:**

The online version of this article (doi:10.1186/s12940-017-0292-7) contains supplementary material, which is available to authorized users.

## Background

Mitochondria are cellular organelles specialized in energy production and produce the majority of intracellular reactive oxygen species (ROS), which are continually generated as toxic by-products by the electron transport chain (ETC). There is a fine balance in ROS signalling maintained by the redox environment. ROS production may be altered as a consequence of exposure to particulate matter (PM). Oxidative stress can occur both when the intracellular and/or intramitochondrial environments are either highly reduced or highly oxidized [1]. Mitochondrial DNA (mtDNA) repairs DNA damage less efficiently compared to nuclear DNA, making it susceptible to ROS and environmental toxicants such as PM [2]. Accumulation of mtDNA damage can cause disturbed mtDNA replication and elimination of damaged mtDNA, and in turn lead to decreased levels of mtDNA [3, 4]. Furthermore, components of the mitochondrial membrane rich in unsaturated fatty acids, such as cardiolipin, are especially sensitive to peroxidation by ROS, resulting in reactive aldehydes which can further damage mitochondrial structures [3, 5]. Increased levels of mtROS or/and accumulation of mitochondrial DNA damage may ultimately lead to programmed cell death [6]. Moreover, oxidative stress and mitochondrial dysfunction are linked with several age-related diseases such as diabetes, cancer, cardiovascular, and neurodegenerative diseases [7–10].

Here, we explored sex-specific associations of PM exposure on expression of mitochondrial associated genes. Furthermore, a pathway analysis was performed on genome wide transcriptome data to investigate whether mitochondrial pathways are highly affected by air pollution exposure. This hypothesis-generating approach identified sex-specific mitochondrial related genes associated with short- and medium-term PM_10_ exposure that were analysed further in a validation study by means of real-time quantitative PCR (qRT-PCR).

## Methods

### Study design

As our study aim was to investigate the association of short- and medium-term PM_10_ exposure with mitochondrial-associated transcriptomic responses in peripheral blood, we performed sex-stratified microarray analyses in a discovery cohort of 98 adults. At gene level, we specifically investigated associations between PM_10_ exposure and expression of 1064 genes listed in the “Human MitoCarta2.0” inventory [11, 12] which are known to encode proteins with mitochondrial localization. Furthermore, we performed pathway analyses on all 15,589 measured transcripts. Based on the gene level and pathway level analyses, we selected 13 MitoCarta genes contributing to top ranked mitochondrial pathways for validation by means of qRT-PCR in an independent validation cohort (*n* = 169). To substantiate the mitochondrial response to air pollution exposure, we further investigate the link between PM_10_ exposure and mtDNA content in peripheral blood in the validation cohort.

### Study population

#### Discovery cohort

The original study population is part of the first Flemish Environment and Health Survey (FLEHS I) and consisted of 398 individuals from eight different regions of residence in Flanders (Belgium) [13]. Inclusion criteria were living in the region of Flanders >5 years, age 50 till 65 years and being able to complete questionnaires in Dutch. Informed consent was obtained from all subjects. Sampling took place between September 2004 and June 2005. Participants donated a blood and urine sample, body height and weight were measured in a standardised way. Demographic data, life style factors and health parameters were provided through an extensive self-assessment questionnaire. A subset of 98 non-smokers was selected for whole genome microarray analysis. The selection procedure was previously described by Vrijens et al. [14].

#### Validation cohort

To validate a selection of MitoCarta genes identified as being associated to PM_10_ exposure in the discovery cohort, we measured whole blood gene expression levels using qRT-PCR in an independent study population of 169 subjects being part of the third Flemish Environment and Health Survey (FLEHS III). Additionally, mtDNA content was determined in peripheral blood of 150 individuals. Inclusion criteria and data collection were similar as for FLESH I. Informed consent was obtained from all participants. The sampling campaign lasted from May until November 2014.

### RNA isolation

Total RNA was extracted from 2.5 ml of whole blood in vacutainers using the Paxgene Blood RNA system (PreAnalytiX, Qiagen, Hilden, Germany), according to the manufacturer’s guidelines. A globin reduction assay (GLOBINclear™ Kit by Ambion, Austin, USA) was performed to remove hemoglobin mRNA from samples assessed in microarray analyses. RNA purity was measured spectrophotometrically and RNA integrity was checked using the BioAnalyzer (Agilent, Palo Alto, USA). Labelled samples were assessed for specific activity and dye incorporation.

### Microarray preparation, hybridization and preprocessing

From each sample of the discovery cohort, 0.2 μg total RNA was used to synthesize fluorescent cyanine-3-labeled cRNA following the Agilent one-color Quick-Amp labelling protocol (Agilent Technologies). Samples were hybridized on Agilent Human Whole Genome 4x44K microarrays (design ID 014850). Microarrays signals were detected with an Agilent G2505C DNA Microarray Scanner (Agilent Technologies). Raw data were entered in an in-house developed quality control pipeline in R software applying following preprocessing steps: local background correction, omission of controls, flagging of bad quality spots (based on the size of the spot, the number of pixels per spot, the mean vs. median ratio of the pixel intensity, intensity of spot is not above background, and/or saturation of the spot), and spots with too low intensity, log_2_-transformation and quantile normalization. Information about the flagging and the R-scripts of the pipeline are available at https://github.com/BiGCAT-UM/arrayQC_Module. Further preprocessing included the omission of probes showing >30% flagged data, merging of replicate probes based on median, and the imputation of missing values using K-nearest neighbor imputation (K = 15). If multiple probes represent the same gene, the probe with the largest interquartile range was selected. The final dataset consisted of 15,589 unique Entrez Gene IDs.

### Exposure assessment

PM_10_ and PM_2.5_ exposure (μg/m^3^) concentrations were modelled using a spatial temporal interpolation method (Kriging) [15] for each participants’ residential address in combination with a dispersion model. The interpolation method uses land-cover data obtained from satellite images (CORINE land-cover data set) and pollution data collected from a governmental stationary monitoring network. Overall model performance was evaluated by leave-one-out cross-validation including 58 and 34 monitoring points for PM_10_ and PM_2.5_ respectively. Validation statistics of the interpolation tool explained >70% of the temporal variability for hourly and annual PM_10_ and PM_2.5_ averages in the Flemish Region of Belgium [16]. Coupled with a dispersion model (Immission Frequency Distribution Model, IFDM) [16, 17] that uses emissions from point sources and line sources, this model chain provides PM values in a dense irregular receptor grid. Previous studies conducted a thorough intercomparison of different models currently in use for regulatory purposes in Europe including IFDM [18–22]. To explore potentially critical exposure windows, we averaged residential one week exposure as a proxy for recent exposure, one month exposure as a proxy for medium-term exposure, two-year exposure as a proxy for long-term exposure. Note that PM_2.5_ exposure estimates were only available for the validation cohort. The Belgian Royal Meteorological Institute provided meteorological data consisting of mean daily air temperature and relative humidity. Apparent temperature was calculated [23, 24] and averaged over the same exposure time window as PM_10_.

### Real-time quantitative PCR (qRT-PCR)

For the validation cohort, total RNA was reverse transcribed to cDNA using the GoScript Reverse Transcription System (Promega, Madison, WI, USA). Gene expression was measured in a 10 μl PCR reaction consisting of 2 μL of a 5 ng/μL dilution of cDNA, TaqMan Fast Advanced Master Mix (Life Technologies, Foster City, CA, USA) and PrimeTimeTM assay (Integrated DNA Technologies, Coralville, IA, USA). Samples were analyzed in triplicate with a 7900HT Fast Real-Time PCR system (Life Technologies, Foster City, CA, USA) applying standard cycling conditions. SDS 2.3 provided threshold cycle (*C*_*p*_) values which were further processed to normalized relative gene expression values with qBase plus (Biogazelle, Zwijnaarde, Belgium). Replicates were included if the difference in C_*p*_ values was <0.5. *HPRT*, *IPO8* and *YWHAZ* were used for data normalization.

### DNA extraction and measurement of mtDNA content

For the validation cohort, DNA was isolated from peripheral blood using the QIAmp DNA Mini Kit (QIAGEN GmbH, Hilden, Germany), following the manufacturer’s guidelines. The quantity and purity of the extracted DNA were determined using a Nanodrop spectrophotometer (ND-1000; Isogen Life Science B.V., De Meern, the Netherlands). The DNA samples were diluted to 2.4 ng/μL. MtDNA was measured by calculating the relative ratio of two mitochondrial sequences [*MT-ND1* and mitochondrial forward primer from nucleotide 3212 and reverse primer from nucleotide 3319 (*MTF3212/R3319*)] to a single housekeeping nuclear gene (*RPLP0*)] measured using a qPCR assay [25]. qPCR was performed using 2.5 μl extracted DNA and 7.5 μl master mix containing Fast SYBR Green dye 2× (Applied Biosystems, Inc., Foster City, California), forward and reverse primers diluted to 300 nM per well, and RNase-free water. Samples were run in triplicate. Each 384-well plate contained 6 interrun calibrators and 2 no-template controls. qPCR was performed using the 7900HT Fast Real-Time PCR System (Life Technologies, Foster City, CA, United States) with following thermal cycling profile: 20 s at 95 °C, followed by 40 cycles of 1 s at 95 °C and 20 s at 60 °C. A melting curve analysis was performed at the end of each run to confirm the absence of nonspecific products. Replicates were included if the difference in *C*_*p*_ values was <0.5. qBase software (Biogazelle, Zwijnaarde, Belgium) was used to normalize *C*_*p*_ values of the two mtDNA sequences relative to the nuclear gene and to correct for run-to-run differences [26].

### Data analysis

Statistical analyses were carried out using SAS software (version 9.3, SAS Institute Inc., Cary, NC, USA). Continuous data were presented as mean and 10th–90th percentiles and categorical data as percentages and frequencies.

#### Discovery cohort

For each gene, a multivariable linear regression was fitted to investigate the association between log_2_-transformed gene expression levels and PM_10_ exposure estimates (short- and medium-term exposure). Previous epidemiological studies reported that environmental stressors have sex-specific immunological responses, with women being more susceptible to smoking than men [27, 28]. Thereupon, in this study we performed sex-stratified analyses to explore both sex-specific and non-specific PM-induced effects and we adjusted for age, body mass index (BMI), socio-economic status (lower secondary or less, higher secondary, higher education), season (medium [April–May, September–November] or cold [December–March]), time of blood sampling (between 08.20 am and 02.30 pm), apparent temperature and microarray batch number (3 scan dates) to correct for batch effects. Of the 15,589 measured genes, 1064 were “Human MitoCarta2.0” genes [11, 12] which are known to encode proteins with mitochondrial localization. Firstly, because of the specific interest in mitochondria, we performed Human MitoCarta gene-wide association scan, with *p*-values adjusted for multiple testing (for the 1064 genes) by controlling the Benjamini-Hochberg (BH) false discovery rate at 5%. FDR adjusted *p*-values are referred to as *q*-values. Secondly, we performed pathway analyses. Of the 15,589 genes, genes with unadjusted *p*-value <0.05 were uploaded into the online overrepresentation analysis (ORA) tool ConsensusPathDB (http://consensuspathdb.org/) [29] developed at the Max Planck Institute for Molecular Genetics, to identify processes altered by PM_10_ exposure. Pathways with a *p*-value <0.05 were considered significant.

#### Validation cohort

Based on the results of the pathway analyses, 7 MitoCarta genes with a *q*-value <0.25 (4 in association with short-term exposure and 3 in association with medium-term exposure), contributing to the top 15 ranked pathways/GO terms and with a well-known functional role within mitochondria were selected in women for validation by qRT-PCR. For men, 6 MitoCarta genes (unadjusted *p*-value <0.05) in relation to medium-term exposure and contributing to the top 15 ranked mitochondrial pathways were selected for validation. We examined the association of expression levels measured by qRT-PCR of the 13 selected genes and of mtDNA content with short-, medium-, and long-term PM_10_ and PM_2.5_ exposure. We adjusted for age, BMI, socio-economic status, smoking, temperature and time as well as season of blood sampling, white blood cell (WBC) count and percentage of neutrophils. Residuals were plotted to check whether significance was driven by outlying gene expression values. Over different time windows, for PM_10_ and PM_2.5_ separately, the BH multiple testing method was applied to correct for the false discover rate (FDR). For the validation cohort, a *q*-value <0.05 was considered significant.

## Results

The characteristics of the discovery and validation cohort are listed in Table 1 for women and men separately. All participants were of European origin. Both cohorts did not differ in the distribution of age and BMI. For both cohorts, age ranged between 50 and 65 years. BMI averaged (range) 26.6 (20.9–38.5) kg/m^2^ in the discovery cohort and 25.8 (16.8–39.4) kg/m^2^ in the validation cohort. Overall, short- and medium-term PM_10_ exposure estimates were higher in the discovery cohort compared to the validation cohort. In the discovery cohort, more subjects were recruited during the cold period of the year compared to the validation cohort (81.6 vs 39.7%). The discovery cohort consisted only of non-smokers, whilst the validation cohort included 21 (12.4%) smokers. In the validation cohort a higher percentage of participants (53.3%) had a high socio-economic status compared to 28.6% in the discovery cohort.

**Table 1:** Descriptive characteristics for women and men of the discovery and validation cohort

Characteristics	Discovery cohort (2004–2005)		Validation cohort (2012–2015)	
*Personal*	Women (*n* = 50)	Men (*n* = 48)	Women (*n* = 94)	Men (*n* = 75)
Age, years	57.8 [51.2–63.1]	58.0 [51.5–64.0]	58.1 [52.6–63.2]	58.0 [52.5–63.6]
BMI, kg/m^2^	25.8 [22.1–31.1]	27.4 [23.0–31.4]	25.5 [20.4–32.8]	26.1 [21.6–30.9]
WBC, cells/mcL			6965 [5170–9360]	6948 [5200–9270]
Socio-economic status				
Low	28 (56.0)	20 (41.7)	23 (56.0)	14 (18.7)
Medium	7 (14.0)	15 (31.3)	16 (14.0)	26 (34.7)
High	15 (30.0)	13 (27.1)	55 (30.0)	35 (46.7)
Smoking status				
Non-smokers	50 (100.0)	48 (100.0)	80 (85.1)	68 (90.7)
Smokers	-	-	14 (14.9)	7 (9.3)
Season of blood sampling				
Cold (October–March)	40 (80.0)	40 (83.3)	40 (42.6)	27 (36.0)
Warm (April–September)	10 (20.0)	8 (16.7)	54 (57.4)	48 (64.0)
Time of blood sampling				
< 12 pm	44 (88.0)	41 (85.4)	7 (7.5)	0 (0.0)
12 pm–3 pm	6 (12.0)	7 (14.6)	25 (26.6)	20 (26.7)
3 pm–6 pm	0 (0.0)	0 (0.0)	43 (45.7)	32 (42.7)
> 8 pm	0 (0.0)	0 (0.0)	19 (20.2)	23 (30.7)
*Exposure*				
Short-term^a^ PM_10_, μg/m^3^	29.3 [18.9–41.3]	30.6 [20.4–41.4]	19.6 [13.0–26.9]	17.8 [12.6–24.0]
Short-term^a^ PM_2.5_, μg/m^3^	-	-	12.8 [5.4–26.0]	11.3 [5.8–17.7]
Medium-term^b^ PM_10_, μg/m^3^	29.7 [24.2–40.5]	31.5 [25.8–40.6]	19.7 [13.8–26.7]	17.5 [13.9–23.8]
Medium-term^b^ PM_2.5_, μg/m^3^	-	-	12.7 [7.4–18.8]	10.8 [7.6–16.9]
Long-term^c^ PM_10_, μg/m^3^	26.0 [21.4–30.2]	25.7 [21.4–30.1]	24.2 [21.3–27.7]	23.1 [20.8–25.8]
Long-term^c^ PM_2.5_, μg/m^3^	17.8 [15.5–20.5]	17.6 [15.6–20.3]	16.0 [14.9–17.5]	15.5 [14.6–16.5]
Week AT, °C	5.8 [−1.3–11.9]	3.4 [−1.5–11.6]	15.4 [12.7–17.3]	15.2 [12.7–17.3]
Month AT, °C	7.1 [0.6–14.1]	4.7 [0.6–11.2]	15.3 [13.2–16.5]	15.3 [13.2–17.2]

^a^Week before blood sampling

^b^Month before blood sampling

^c^Two-year averages

Data are number (%) or mean [10th-90th percentile]. *AT* apparent temperature

### Gene level analysis

For short- and medium-term exposure, volcano plots of all measured transcripts are presented for both sexes in (Additional file 1: Figure S1). Overall responses to PM_10_ exposure seem to differ between women and men.

Table 2 lists the top 10 Human MitoCarta genes and their corresponding fold changes for an increase in short-term PM_10_ exposure of 10 μg/m^3^ for women and men. For women, 8 genes were significantly associated with short-term PM_10_ exposure. The top significant gene for women, *POLG*, encoding the catalytic subunit of the mtDNA polymerase, was downregulated. For men, no significant genes after correction for multiple testing were found. The top ranked gene was *IDI1* required in the mevalonate pathway. None of the 8 significant genes in women were in the top 100 of men.

**Table 2:** Top 10 most strongly associated Human MitoCarta genes with short-term PM_10_ exposure for women and men

Gene symbol	Gene name	FC (95% CI)	*P*-value	*Q*-value
Women				
*POLG*	polymerase (DNA) gamma, catalytic subunit	0.83 (0.78, 0.90)	1.31E-05	0.01
*MRPL38*	mitochondrial ribosomal protein L38	0.83 (0.76, 0.90)	1.08E-04	0.04
*MRPL16*	mitochondrial ribosomal protein L16	0.83 (0.77, 0.91)	1.46E-04	0.04
*OGG1*	8-oxoguanine DNA glycosylase	0.81 (0.73, 0.89)	1.48E-04	0.04
*ECHS1*	enoyl-CoA hydratase, short chain, 1, mitochondrial	0.87 (0.81, 0.93)	1.92E-04	0.04
*GTPBP3*	GTP binding protein 3 (mitochondrial)	0.82 (0.74, 0.90)	1.99E-04	0.04
*ECI2*	enoyl-CoA delta isomerase 2	0.84 (0.77, 0.92)	2.86E-04	0.04
*ETHE1*	ETHE1, persulfide dioxygenase	0.86 (0.79, 0.92)	3.23E-04	0.04
*POLRMT*	polymerase (RNA) mitochondrial	0.86 (0.80, 0.93)	4.91E-04	0.06
*BOLA1*	bolA family member 1	0.87 (0.81, 0.93)	5.64E-04	0.06
Men				
*IDI1*	isopentenyl-diphosphate delta isomerase 1	1.20 (1.08, 1.33)	1.31E-03	0.97
*CKMT1A*	creatine kinase, mitochondrial 1A	1.30 (1.10, 1.53)	4.09E-03	0.97
*PGS1*	phosphatidylglycerophosphate synthase 1	1.30 (1.10, 1.54)	4.32E-03	0.97
*GNG5*	G protein subunit gamma 5	1.12 (1.04, 1.21)	7.37E-03	0.97
*TMBIM4*	transmembrane BAX inhibitor motif containing 4	1.12 (1.03, 1.22)	1.01E-02	0.97
*CHCHD6*	coiled-coil-helix-coiled-coil-helix domain containing 6	1.11 (1.03, 1.19)	1.30E-02	0.97
*CHCHD8*	coiled-coil-helix-coiled-coil-helix domain containing 8	1.17 (1.04, 1.32)	1.44E-02	0.97
*COX6A2*	cytochrome c oxidase subunit 6A2	1.26 (1.06, 1.50)	1.46E-02	0.97
*RPL34*	ribosomal protein L34	0.83 (0.72, 0.96)	1.49E-02	0.97
*DHRS7B*	dehydrogenase/reductase 7B	1.15 (1.03, 1.28)	1.58E-02	0.97

*FC* fold change calculated for an increase in PM_10_ of 10 μg/m^3^

Table 3 list the top 10 mitochondria-localized genes based on their *p*-value and there corresponding fold changes for an increase in medium-term PM_10_ exposure of 10 μg/m^3^ for women and 10 highest ranked genes for men. *ALDH7A1* (*q*-value: 0.21) and *MRPS15* (*q*-value: 0.47) were the top ranked genes for women and men respectively.

**Table 3:** Top 10 most strongly associated Human MitoCarta genes with medium-term PM_10_ exposure for women and men

Gene symbol	Gene name	FC (95% CI)	*P*-value	*Q*-value
Women				
*ALDH7A1*	aldehyde dehydrogenase 7 family member A1	0.70 (0.54, 0.90)	2.36E-04	0.21
*TIMM17B*	translocase of inner mitochondrial membrane 17 homolog B (yeast)	1.05 (0.97, 1.13)	3.90E-04	0.21
*GOT2*	glutamic-oxaloacetic transaminase 2	0.92 (0.68, 1.24)	8.06E-04	0.23
*PDP2*	pyruvate dehyrogenase phosphatase catalytic subunit 2	1.04 (0.95, 1.15)	9.34E-04	0.23
*GLS*	glutaminase	0.79 (0.62, 1.00)	1.40E-03	0.23
*FXN*	frataxin	0.88 (0.81, 0.97)	1.47E-03	0.23
*HINT2*	histidine triad nucleotide binding protein 2	0.97 (0.88, 1.06)	2.46E-03	0.23
*UQCRH*	ubiquinol-cytochrome c reductase hinge protein	0.97 (0.86, 1.09)	2.56E-03	0.23
*CPT2*	carnitine palmitoyltransferase 2	1.03 (0.94, 1.12)	2.59E-03	0.23
*TRUB2*	TruB pseudouridine synthase family member 2	0.98 (0.89, 1.07)	3.25E-03	0.23
Men				
*MRPS15*	mitochondrial ribosomal protein S15	1.23 (1.10, 1.38)	1.14E-03	0.47
*CLPB*	ClpB homolog, mitochondrial AAA ATPase chaperonin	1.36 (1.15, 1.61)	1.15E-03	0.47
*SLC25A29*	solute carrier family 25 member 29	1.64 (1.24, 2.16)	1.32E-03	0.47
*BCKDK*	branched chain ketoacid dehydrogenase kinase	1.24 (1.09, 1.43)	3.37E-03	0.55
*STOML1*	stomatin-like 1	1.25 (1.08, 1.44)	4.55E-03	0.55
*ADCK1*	aarF domain containing kinase 1	1.33 (1.10, 1.61)	5.22E-03	0.55
*SLC25A40*	solute carrier family 25 member 40	0.66 (0.50, 0.87)	6.23E-03	0.55
*ALDH7A1*	aldehyde dehydrogenase 7 family member A1	1.58 (1.16, 2.16)	6.35E-03	0.55
*MDH2*	malate dehydrogenase 2	1.29 (1.09, 1.54)	6.67E-03	0.55
*ALDH1B1*	aldehyde dehydrogenase 1 family member B1	1.36 (1.10, 1.69)	7.33E-03	0.55

*FC* fold change calculated for an increase in PM_10_ of 10 μg/m^3^

### Overrepresentation analysis

Sex-specific PM_10_ effects were further explored by overrepresentation analyses (ORA). Tables 4 and 5 represent the top 15 significant pathways, with at least 15 measured genes and a total gene size of at most 150 genes, related to, respectively, short- and medium-term PM_10_ exposure for both sexes. For pathways with the same contributing genes, only the most significant pathway is shown. Mitochondrial pathways, containing mainly MitoCarta genes, are marked with an asterisk. Human MitoCarta genes are indicated in bold font.

**Table 4:** Top 15 significant pathways associated with short-term exposure

Pathway	Effective/ total size	# ↓ genes	Contributing genes (#)^§^	*P*-value
Women				
IL12-mediated signaling events	57/67	33	*TBX21*↓;*CD247*↓;*MAPK14*↑;*CCL4*↓;*CCR5↓(20)*	1.8E-06
role of mef2d in t-cell apoptosis	28/31	25	*CD247*↓;*ZAP70*↓;*FYN*↓;*LAT*↓;*CD3E*↓;*CABIN1*↓;*PPP3CC*↓;*LCK*↓;*PLCG1*↓;*CAPN2*↓;*CD3D↓(11)*	1.2E-04
T cell receptor signaling pathway	96/104	52	*CD247*↓;*PDCD1*↓;*MAPK14*↑;*ZAP70*↓;*FYN↓(22)*	8.0E-04
Ribosome	128/135	100	*RPLP2*↓;***MRPL16*** **↓;** *RPL36*↓;*RPL35*↓;*RPL18↓(27)*	8.6E-04
Natural killer cell mediated cytotoxicity	104/134	50	*CD247*↓;*HCST*↓;*ZAP70*↓;*FYN*↓;*LAT↓(23)*	1.0E-03
Downstream signaling in naïve CD8+ T cells	52/71	33	*CD247*↓;*IL2RB*↓;*EOMES*↓;*CD8A*↓;*CD3E*↓;*PRF1*↓;*TNFRSF4*↓;*PTPN7*↓;*STAT4*↓;*GZMB*↓;*BRAF*↑;*MAPK3*↑;*CD3D*↓;*MAPK1↑(14)*	1.4E-03
Formation of a pool of free 40S subunits	94/151	71	*RPLP2*↓;*RPL36*↓;*RPL35*↓;*RPL18*↓;*RPLP1↓(21)*	1.5E-03
Primary immunodeficiency	32/36	24	*ZAP70*↓;*ADA*↓;*RFXAP*↓;*DCLRE1C*↓;*CD8A*↓;*CD3E*↓;*CD19*↓;*ICOS*↓;*LCK*↓;*CD3D↓(10)*	2.0E-03
TCR signaling in naïve CD8+ T cells	54/58	35	*RASGRP2*↓;*CD247*↓;*ZAP70*↓;*FYN*↓;*LAT*↓;*CARD11*↓;*CD8A*↓;*CBL*↑;*RASGRP1*↓;*CD3E*↓;*PRF1*↓;*LCK*↓;*PLCG1*↓;*CD3D↓(14)*	2.0E-03
Mitochondrial translation (elongation)*	84/85	72	***MRPL38*** **↓;** ***MRPL16*** **↓;** ***MRPS9*** **↓;** ***MRPL4*** **↓;** ***MRPS26↓*** *(19)*	2.1E-03
NF-κB signaling pathway	86/91	43	*PARP1*↓;*TRAF2*↓;*ZAP70*↓;*CCL4*↓;*TRAF5↓(19)*	2.8E-03
Immunoregulatory interactions between a Lymphoid and a non-Lymphoid cell	64/132	41	*CD247*↓;*HCST*↓;*ICAM4*↑;*ITGB7*↓;*CD8A*↓;*CD96*↓;*KIR2DL2*↓; *CD3E*↓;*CD19*↓;*KIR3DL1*↓;*KLRD1*↓;*KIR3DL2*↓;*ITGB1*↓;*ITGA4*↓;*CD3D↓(15)*	4.2E-03
Busulfan Pathway, Pharmacodynamics	30/36	16	***BNIP3*** **↓;** *CHEK2*↓;***BCL2L1*** **↑;** ***MLH1*** **↓;** *FMO5*↑;*LTBR*↑;*GSTP1*↓;*GGT1*↑;*MPG↓(9)*	4.5E-03
Cell cycle	116/124	63	*ZBTB17*↓;*ANAPC1*↓;*CDK4*↓;*MCM7*↓;*CHEK2↓(23)*	4.6E-03
Downregulation of SMAD2/3:SMAD4 transcriptional activity	20/21	10	*PARP1*↓;*NCOR2*↓;*RPS27A*↓;*SMAD3*↓;*UBB*↑;*PPM1A*↑;*NEDD4L↑(7)*	4.6E-03
Men				
Meiotic recombination	53/64	3	*HIST1H3C*↑;*HIST1H2B*↑;*HIST2H3A*↑;*HIST1H2B*↑;*HIST3H2B↑(22)*	1.3E-12
Cytokine Signaling in Immune system	156/198	49	*STAT2*↑;*IRS2*↑;*CSH1*↑;*PELI1*↑;*EIF4E3↑(24)*	1.7E-04
Osteoclast differentiation	120/131	25	*CYBA*↑;*FCGR3B*↑;*FOSL2*↑;*GRB2*↑;*IFNAR1↑(20)*	2.0E-04
Phagosome	135/155	35	*ATP6V1E1*↑;*CD14*↑;*CYBA*↑;*CLEC4M*↑;*FCAR↑(21)*	3.7E-04
GMCSF-mediated signaling events	36/41	10	*FOS*↑;*PRKACA*↑;*STAT5B*↑;*OSM*↑;*GRB2*↑;*MAP2K1*↑;*LYN*↑;*STAT5A*↑;*MAPK3↑(9)*	6.0E-04
Legionellosis	51/55	13	*CASP1*↑;*CD14*↑;*CXCL3*↑;*HBS1L*↑;*HSPA1A*↑;*IL1B*↑;*MYD88*↑;*RAB1A*↑;*TLR2*↑;*TLR4*↑;*VCP↑(11)*	6.1E-04
zTuberculosis	151/179	46	*CEBPB*↑;*CTSD*↑;*CD14*↑;*RHOA*↑;*CLEC4M↑(22)*	6.9E-04
Growth hormone receptor signaling	17/20	1	*IRS2*↑;*CSH1*↑;*SOCS3*↑;*STAT5B*↑;*LYN*↑;*STAT5A↑(6)*	6.9E-04
Oncostatin_M	37/40	9	*CEBPB*↑;*FOS*↑;*OSMR*↑;*SOCS3*↑;*JUNB*↑;*STAT5B*↑;*OSM*↑;*GRB2*↑;*MAPK3↑(9)*	7.5E-04
IL3 Signaling Pathway	47/40	13	*MAP2K1*↑;*LYN*↑;*GRB2*↑;*PRKACA*↑;*HCK*↑;*MAPK3*↑;*STAT5B*↑;*VAV1*↑;*FOS*↑;*STAT5A↑(10)*	1.2E-03
Endogenous Toll-like receptor signaling	25/27	5	*CD14*↑;*TLR4*↑;*RHOA*↑;*TLR2*↑;*TLR1*↑;*MYD88*↑;*TLR6↑(7)*	1.2E-03
Salmonella infection	73/86	20	*MYD88*↑;*ACTB*↑;*RAB7*↑;*FOS*↑;*CASP1*↑;*CXCL3*↑;*ARPC4*↑;*IFNGR2*↑;*CD14*↑;*MYH14*↑;*TLR4*↑;*MAPK3*↑;*IL1B↑(13)*	1.4E-03
Kit receptor signaling pathway	57/59	11	*MAP2K1*↑;*LYN*↑;*GRB2*↑;*GRB7*↑;*JUNB*↑;*MAPK3*↑;*STAT5A*↑;*STAT5B*↑;*RPS6KA1*↑;*VAV1*↑;*FOS↑(11)*	1.6E-03
Toll-like receptor signaling pathway	84/102	18	*IL1B*↑;*MAPK3*↑;*CD14*↑;*TLR6*↑;*MAP2K1*↑;*IRF7*↑;*TLR1*↑;*IKBKE*↑;*TLR2*↑;*IFNAR1*↑;*TLR4*↑;*FADD*↑;*FOS*↑;*MYD88↑(14)*	1.8E-03
Cytoplasmic Ribosomal Proteins	85/88	73	***RPS18*** **↓;** *RPL27*↓;*RPL27A*↓;***RPL10A*** **↓;** *RPL19*↓;*RPL18*↓;*RPLP1*↓;***RPL34*** **↓;** *RPS8*↓;*RPL13A*↓;*RPS6KA1*↑;*RPL11*↓;*RPS29*↓;*RPS27↓(14)*	2.0E-03

# ↓ Number of down-regulated genes. ^§^If more than 15 contributing genes only the top 5 is given. Mitochondrial pathways are marked with an asterisk and MitoCarta genes are indicated in bold type. IL: Interleukin; MEF2D: myocyte enhancer factor 2D; SMAD2,3,4: SMAD family member 2,3,4; GMCSF: Granulocyte-macrophage colony-stimulating factor

**Table 5:** Top 15 significant pathways associated with medium-term exposure

	Pathway	Effective/ total size	# ↓ genes	Contributing genes (#)^§^	*P*-value
Women					
	RNA Polymerase I Chain Elongation	79/98	25	*HIST2H2A*↑;*HIST1H2A*↑;*HIST1H4E*↑;*HIST1H2A*↑;*POLR1E↓(24)*	1.7E-04
	Meiosis	63/77	20	*HIST2H2A*↑;*HIST1H2A*↑;*HIST1H4E*↑;*HIST1H2A*↑; *HIST1H4I↑(20)*	3.0E-04
	RMTs methylate histone arginines	64/74	23	*HIST2H2A*↑;*RBBP7*↓;*HIST1H2A*↑;*HIST1H2A*↑; *HIST1H4E↑(20)*	3.8E-04
	RNA Polymerase I Transcription	100/121	35	*HIST2H2A*↑;*RBBP7*↓;*HIST1H2A*↑;*HIST1H4E*↑; *HIST1H2A↑(27)*	5.7E-04
	Mitochondrial translation (termination)*	84/85	25	***MRPL50*** **↓;** ***MRPL22*** **↑;** ***MRPL30*** **↑;** ***MRPL47*** **↑;** ***MRPL54↑*** *(23)*	1.1E-03
	phospholipids as signalling intermediaries	30/36	12	*EDG1*↓;*MAP2K1*↓;*ITGAV*↓;*SOS1*↑;*SRC*↑;*PDGFA*↑;*PIK3CA*↓;*ASAH1*↑;*PDPK1*↑;*HRAS*↑;*ADCY1↓(11)*	1.9E-03
	Meiotic recombination	53/64	15	*HIST2H2A*↑;*HIST1H2A*↑;*HIST1H4E*↑;*HIST1H2A*↑;*HIST1H4I↑(16)*	2.1E-03
	Respiratory electron transport, ATP synthesis by chemiosmotic coupling, and heat production by uncoupling proteins.*	99/113	23	***UQCRH*** **↑;** ***ATP5L*** **↓;** ***NDUFA13*** **↑;** ***COX7C*** **↑;** ***UCP3↑*** *(25)*	2.5E-03
	Meiotic synapsis	40/48	8	*HIST1H4E*↑;*HIST1H4I*↑;*HIST1H2A*↑;*HIST4H4*↑;*RAD21*↓;*HIST1H4H*↑;*ATR*↓;*HIST2H4B*↑;*HIST1H4J*↑;*BRCA1*↑;*HIST1H4K*↑;*REC8L1*↑; *HIST1H2B↑(13)*	2.6E-03
	Sirtuin 1 negatively regulates rRNA Expression	59/76	18	*HIST1H4H*↑;*HIST2H2A*↑;*HIST1H4I*↑;*HIST1H2A*↑; *HIST1H2A↑(17)*	2.7E-03
	Condensation of Prophase Chromosomes	64/77	17	*HIST2H2A*↑;*HIST1H2A*↑;*HIST1H4E*↑;*HIST1H2A*↑; *HIST1H4I↑(18)*	2.8E-03
	NoRC negatively regulates rRNA expression	95/116	34	*HIST2H2A*↑;*HIST1H2A*↑;*HIST1H4E*↑;*HIST1H2A*↑; *POLR1E↓(24)*	3.0E-03
	RNA Polymerase I, RNA Polymerase III, and Mitochondrial Transcription	133/159	48	*HIST2H2A*↑;*RBBP7*↓;*HIST1H2A*↑;*HIST1H4E*↑;*HIST1H2A↑(31)*	3.2E-03
	Platelet Aggregation (Plug Formation)	28/38	9	*GP1BA*↑;*SOS1*↑;*SRC*↑;*VWF*↑;*RASGRP2*↓;*GP9*↑;*ITGA2B*↑;*PDPK1*↑; *TLN1*↑;*RAP1A↓(10)*	3.7E-03
	Respiratory electron transport*	81/92	17	***UQCRH*** **↑;** ***NDUFA13*** **↑;** ***COX7C*** **↑;** ***FAM36A*** **↑;** ***NDUFA6↑*** *(21)*	3.8E-03
Men					
	3-phosphoinositide biosynthesis	25/29	10	*C17orf38*↑;*PIK4CB*↑;*PIK3R1*↑;*PIP5K3*↓;***PIK4CA*** **↑;** *CDIPT*↑; *PIP5K2B*↑;*PIK3R2↑(8)*	2.0E-04
	superpathway of inositol phosphate compounds	62/71	31	*C17orf38*↑;*PIK4CB*↑;*PIK3R1*↑;*TMEM55A*↓;*PIP5K3*↓;*IHPK2*↑;*SKIP*↑; ***PIK4CA*** **↑;** *CDIPT*↑;*PIP5K2B*↑;*PIK3R2*↑;*OCRL*↓;*HISPPD1↓(13)*	2.8E-04
	TCA cycle*	27/34	7	***MDH2*** **↑;** ***IDH2*** **↑;** ***IDH3G*** **↑;** ***SUCLA2*** **↑;** ***SDHA*** **↑;** ***CLYBL*** **↑;** ***PCK2*** **↑;** ***ACO2↑*** *(8)*	3.7E-04
	Citrate cycle (TCA cycle)*	28/30	9	***MDH2*** **↑;** ***IDH2*** **↑;** ***PC*** **↑;** ***IDH3G*** **↑;** ***SUCLA2*** **↑;** ***SDHA*** **↑;** ***PCK2*** **↑;** ***ACO2↑*** *(8* ***)***	4.8E-04
	PI Metabolism	47/53	19	*C17orf38*↑;*PIK4CB*↑;*PIK3R1*↑;*PIP5K3*↓;*SKIP*↑;*PIK4CA*↑;*PIP5K2B*↑;*ARF1*↑;*PIK3R2*↑;*OCRL↓(10)*	1.3E-03
	IRS-related events triggered by IGF1R	74/93	26	*GBL*↑;*MAP2K2*↑;*FGFR4*↑;*IGF2*↑;*STK11*↑;*PIK3R1*↑;*DOK1*↑; *FGF4*↑;*AKT2*↑;*TYK2*↑;*PIK3R2*↑;*TLR9*↓;*SOS1↓(13)*	1.7E-03
	Warburg Effect*	43/45	15	***PC*** **↑;** ***IDH3G*** **↑;** ***GAPDH*** **↑;** *SLC1A5*↑;***SDHA*** **↑;** *ENO1*↑;*PKM2*↑;***ACO2*** **↑;** *PGD↑(9)*	2.5E-03
	Synthesis of PIPs at the Golgi membrane	15/20	6	*PIK4CB*↑;*PIP5K3*↓;*PIK4CA*↑;*ARF1*↑;*OCRL↓(5)*	2.7E-03
	superpathway of conversion of glucose to acetyl CoA and entry into the TCA cycle	44/52	13	*MDH2*↑;*GCK*↑;*IDH3G*↑;*GAPDH*↑;*SUCLA2*↑;*SDHA*↑;*ENO1*↑;*PKM2*↑; *ACO2↑(9)*	2.9E-03
	Histidine metabolism	23/36	7	***ALDH7A1*** **↑;** ***ALDH1B1*** **↑;** *SLC38A5*↑;*SLC1A4*↑;*SLC1A5*↑; *SLC38A3↑(6)*	4.1E-03
	Ghrelin	31/44	7	*PIK3R1*↑;*DOK1*↑;*AP2M1*↑;*NOS3*↑;*GNAI2*↑;*PRKCE*↓; *RICTOR↓(7)*	4.7E-03
	IL6 signaling pathway	39/43	9	*MAP2K2*↑;*PIK3R1*↑;*HCK*↑;*NR2F6*↑;*TYK2*↑;*PIK3R2*↑;***BCL2L1*** **↑;** *SOS1↓(7)*	4.8E-03
	Cell-Cell communication	95/130	29	*PIK3R1*↑;*ACTN4*↑;*PARD6G*↑;*ACTN3*↑;*PAK1*↑;*SIRPG*↑;*KIRREL2*↑; *DSCAM*↑;*CDH24*↑;*PIK3R2*↑;*CDH3*↑;*CLDN23*↑;*CLDN3*↑;*FLNC↑(14)*	6.0E-03
	Regulation of toll-like receptor signaling pathway	116/142	38	*GBL*↑;*MAP2K2*↑;*PIK3R1*↑;*IKBKE*↑;*SQSTM1↑(16)*	6.6E-03
	Downstream signaling of activated FGFR	127/155	46	*CDKN1A*↑;*GBL*↑;*MAP2K2*↑;*FGFR4*↑;*PIK3R1↑(17)*	7.0E-03

# ↓ Number of down-regulated genes. ^§^ If more than 15 contributing genes only the top 5 is given. Mitochondrial pathways are marked with an asterisk and MitoCarta genes are indicated in bold type. RMTs: arginine methyltransferases; IGF1R: insulin-like growth factor 1; FGFR: fibroblast growth factor receptors

In women, mitochondrial translation was the top ranked mitochondrial pathway associated with short-term PM_10_ exposure of one week before sampling (Table 4). In addition, PM_10_ exposure was associated with the reaction pathway of busulfan and other DNA damaging agents (*p*-value: 0.004), by deregulating the expression of pro-apoptotic (e.g. *BNIP3*, *LTBR* and *BCL2L1*, isoform Bcl-xS), anti-apoptotic (e.g. *BCL2L1,* isoform Bcl-xL), DNA repair (e.g. *MLH1)*, and detoxifying genes (e.g. *GSTP1* and *GGT1*). Of these, *BNIP3, BCL2L1* and *MLH1* encode proteins (partially) localized in the mitochondria [11, 12].

Mitochondrial GO terms associated with short-term PM_10_ exposure included “mitochondrial respiratory chain complex I biogenesis” (*p*-value: 0.001), of which most genes were downregulated, “regulation of mitochondrial membrane permeability” (*p*-value: 0.0007) playing a crucial role in apoptosis and “mitochondrial genome maintenance” (*p*-value: 0.026) including genes important for mitochondrial biogenesis and cardiolipin biosynthesis (e.g. *STOML2*), mtDNA replication (e.g. *POLG*), mitochondria-mediated apoptosis (e.g. *DNAJA3*), and unfolded protein response in the mitochondrial matrix (e.g. *LONP1*). These four Human Mitocarta genes (*q*-value <0.25) were selected for validation in an independent study population. All were downregulated by medium-term PM_10_ exposure, except for *STOML2*.

In men, ORA did not reveal any mitochondrial pathways/GO terms associated with short-term PM_10_ exposure.

**Fig. 1:**
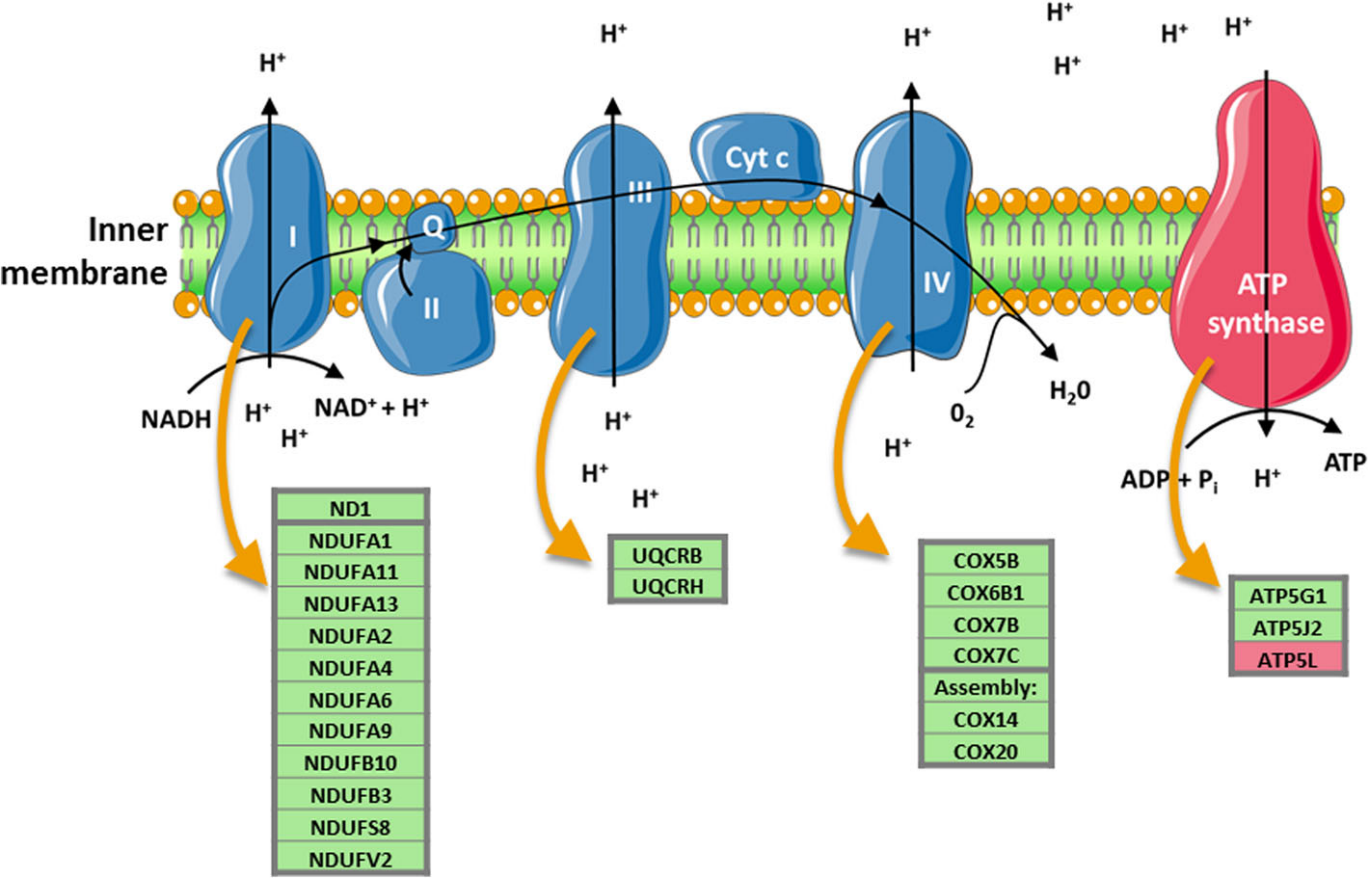
Schematic overview of the mitochondrial respiratory electron transport chain and the genes significantly associated with medium-term PM_10_ exposure per complex in women. Green and red boxes indicate significantly up- and down-regulated genes respectively. Cyt c: cytochrome C

The top 15 significant overrepresented pathways associated to medium-term exposure to PM_10_ of one month before sampling are listed for women and men in Table 5. In women, top significant mitochondrial processes altered by medium-term PM_10_ exposure included mitochondrial translation (*p*-value: 0.001) and the respiratory electron transport chain (*p*-value: 0.004). A more detailed overview of the respiratory chain is given in Fig. 1. All contributing genes in association to PM_10_ were upregulated except for *ATP5L*, a gene encoding a protein of the ATP synthase complex. *NDUFA13*, *UQCRH*, and *COX7C* (*q*-value <0.25) were selected for further validation.

**Fig. 2:**
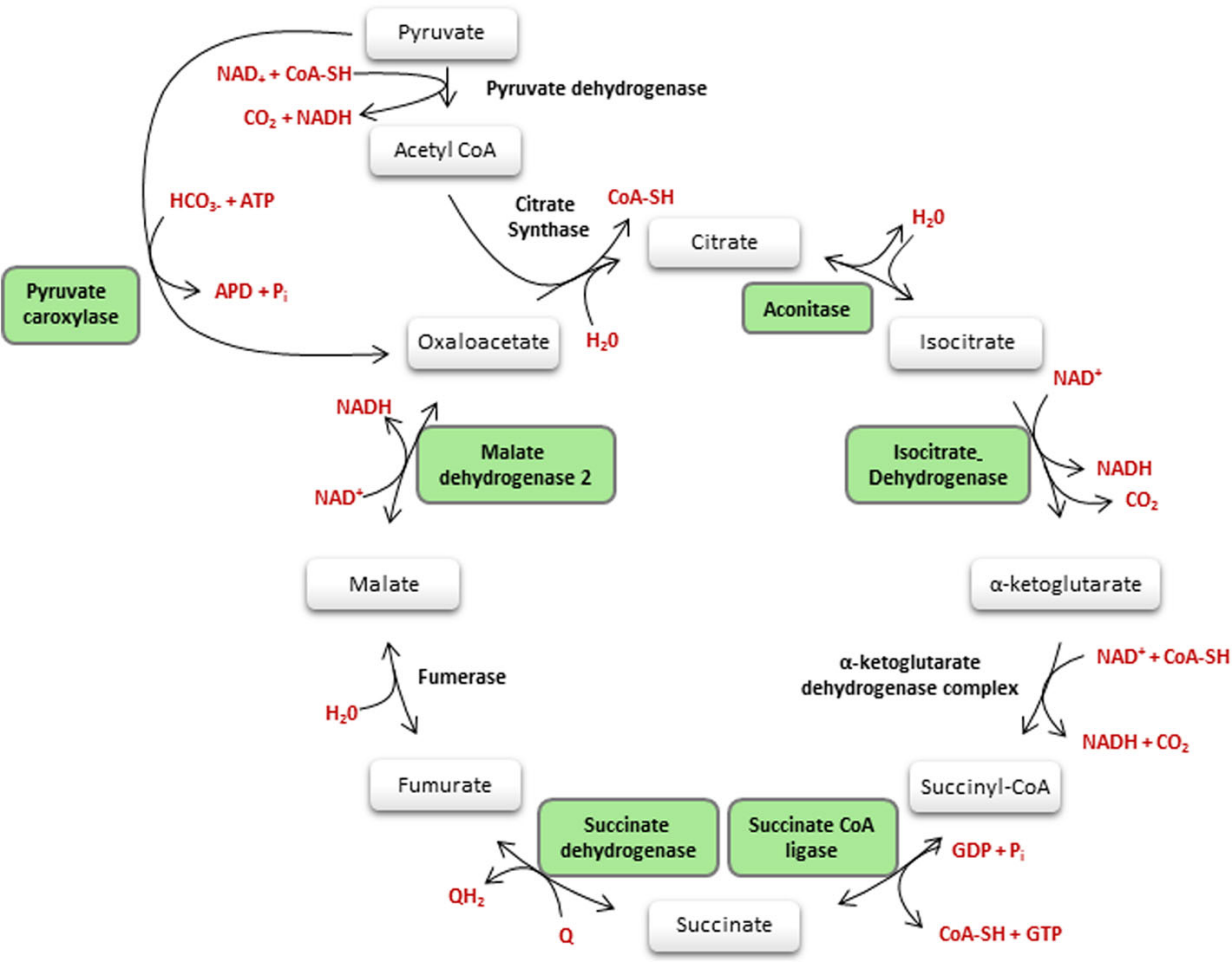
Schematic overview of the TCA cycle. Green boxes indicate significantly up-regulated genes and their corresponding protein in men in association to short-term PM_10_ exposure

For men, the Tri Carbonic Acid (TCA) cycle (*p*-value: 0.0004) was the top mitochondrial pathway associated with medium-term PM_10_ exposure and is represented in Fig. 2. Contributing genes in this pathway were all upregulated. *MDH2*, *IDH2*, *PC*, *SUCLA2*, *SDHA*, and *ACO2* (*p*-value <0.05) were validated in an independent study population. Other significant pathways including contributing Human MitoCarta genes, were 3-phosphoinositide biosynthesis, IL6-signaling pathway, and histidine metabolism. Phosphoinositide 3-kinases (PI3K) are crucial for various general cellular processes, including cell survival and apoptosis. The IL6-signaling pathway contains the MitoCarta gene *BCL2L1* of which the expression was upregulated in men exposed to relatively high medium-term PM_10_ exposure. Furthermore, the expression of some mitochondria-localized aldehyde dehydrogenases (*ALDH7A1*, *ALDH1B1*), participating in the histidine metabolism pathway, was upregulated by medium-term PM_10_ exposure in men.

ConsensuspathDB analyses revealed overrepresented GO terms regarding mitochondrial functioning consistent with the pathway analysis such as the electron transport chain (*p*-value: 0.008) and mitochondrial translational (*p*-value: 0.001) in women and the TCA cycle (*p*-value: 0.009) in men.

### Validation

For the validation cohort, both PM_10_ and PM_2.5_ estimates were available. Results on long-term PM_10_ exposure in the discovery cohort were published in a previous paper [14]. However, analysis of short- and medium-term exposure with microarray data in the discovery cohort and qPCR validation of MitoCarta genes in an independent cohort is novel. Table 6 present the fold changes (95% CI) and *p*-values for the linear association between the 7 MitoCarta genes selected for women in the discovery cohort and short-, medium-, and long-term PM_10_ and PM_2.5_ exposure in women and men of the validation cohort. For women, several of the selected genes were associated with medium- and/or long-term PM exposure. Of the genes contributing to mitochondrial genome maintenance, expression levels of *POLG* and *LONP1* were negatively associated with long-term PM_2.5_ exposure (*q*-value: 0.04 and 0.07 respectively) and *DNAJA3* and *LONP1* were downregulated by medium-term PM_2.5_ exposure (*q*-value: 0.05 and 0.07 respectively). ETC genes were upregulated by PM_10_ and PM_2.5_ for all time windows, however only significantly for the association between long-term PM_2.5_ exposure and *COX7C* and *UQCRH* gene expression (*q*-value <0.05). For men, none of the selected TCA contributing genes could be validated. However, consistent with the observations in women, *LONP1* was negatively associated and *UQCRH* and *NDUFA13* were positively associated with long-term PM_10_ and PM_2.5_ exposure in men of the validation cohort. However, after FDR correction associations in men were not significant.

**Table 6:** Association between expression levels of the selected genes and PM exposure in women (*n* = 94) and men (*n* = 75) of the validation cohort

Sex	Gene symbol	FC (95%CI)	P-val	FC (95%CI)	P-val	FC (95%CI)	P-val	FC (95%CI)	P-val	FC (95%CI)	P-val	FC (95%CI)	P-val
		Short-term PM_10_		Short-term PM_2.5_		Medium-term PM_10_		Medium-term PM_2.5_		Long-term PM_10_		Long-term PM_2.5_	
Women	Mt genome												
	*POLG*	0.76 (0.57,1.00)	0.06	0.75 (0.57,1.00)	0.05	0.65 (0.39,1.10)	0.11	0.57 (0.32,1.01)	0.06	0.90 (0.78,1.03)	0.12	0.62 (0.45,0.86)	0.005^$$^
	*STOML2*	1.13 (0.92,1.37)	0.25	1.11 (0.91,1.35)	0.32	0.95 (0.68,1.34)	0.78	0.95 (0.65,1.39)	0.80	1.03 (0.94,1.13)	0.57	1.21 (0.97,1.51)	0.09
	*DNAJA3*	0.92 (0.81,1.05)	0.23	0.90 (0.79,1.03)	0.13	0.79 (0.63,0.99)	0.04	0.72 (0.56,0.93)	0.01^$^	0.96 (0.90,1.02)	0.22	0.88 (0.76,1.03)	0.11
	*LONP1*	0.84 (0.66,1.09)	0.20	0.84 (0.65,1.08)	0.19	0.64 (0.40,1.00)	0.05	0.55 (0.34,0.90)	0.02^$^	0.91 (0.80,1.03)	0.15	0.70 (0.52,0.93)	0.02^$^
	ETC												
	*COX7C*	1.22 (0.85,1.77)	0.28	1.19 (0.83,1.72)	0.35	1.50 (0.78,2.86)	0.22	1.56 (0.76,3.22)	0.23	1.19 (1.01,1.41)	0.04	1.82 (1.23,2.70)	0.004^$$^
	*UQCRH*	1.32 (0.90,1.93)	0.16	1.29 (0.88,1.89)	0.19	1.29 (0.66,2.49)	0.46	1.42 (0.69,2.94)	0.35	1.14 (0.96,1.37)	0.15	1.81 (1.20,2.71)	0.006^$$^
	*NDUFA13*	1.15 (0.89,1.50)	0.30	1.12 (0.87,1.46)	0.38	1.01 (0.65,1.59)	0.95	1.03 (0.63,1.69)	0.90	1.05 (0.93,1.19)	0.45	1.37 (1.03,1.81)	0.03
Men	Mt genome												
	*POLG*	0.94 (0.86, 1.03)	0.18 ^a^	0.95 (0.87, 1.04)	0.33 ^a^	0.90 (0.77, 1.04)	0.16 ^a^	0.90 (0.77, 1.05)	0.17 ^a^	0.96 (0.92, 1.01)	0.09 ^a^	0.91 (0.82, 1.01)	0.07 ^a^
	*STOML2*	0.96 (0.86, 1.08)	0.52	0.98 (0.88, 1.1)	0.78	0.88 (0.73, 1.06)	0.17	0.88 (0.72, 1.07)	0.19	1.01 (0.96, 1.06)	0.76	0.99 (0.87, 1.12)	0.88
	*DNAJA3*	1.01 (0.92, 1.10)	0.85	1.02 (0.94,1.11)	0.61	1.08 (0.94, 1.24)	0.29	1.08 (0.94, 1.26)	0.29	1.03 (0.99, 1.07)	0.22	1.05 (0.96, 1.16)	0.27
	*LONP1*	0.90 (0.81, 1.11)	0.52 ^b^	0.98 (0.84, 1.14)	0.82 ^b^	0.86 (0.67, 1.11)	0.25 ^b^	0.89 (0.68, 1.15)	0.37 ^b^	0.93 (0.87, 1.00)	0.04 ^b^	0.80 (0.68, 0.94)	0.01 ^b^
	ETC												
	*COX7C*	1.09 (0.85, 1.40)	0.49	1.11 (0.88, 1.41)	0.38	1.17 (0.78, 1.75)	0.46	1.23 (0.81, 1.88)	0.34	1.08 (0.96, 1.21)	0.190	1.23 (0.94, 1.60)	0.14
	*UQCRH*	1.14 (0.92, 1.41)	0.25	1.16 (0.94, 1.44)	0.16	1.39 (0.97, 1.98)	0.07	1.41 (0.97, 2.04)	0.07	1.12 (1.01, 1.24)	0.04	1.32 (1.04, 1.69)	0.03
	*NDUFA13*	1.03 (0.93, 1.15)	0.58	1.05 (0.95, 1.17)	0.36	1.14 (0.96, 1.35)	0.14	1.13 (0.95, 1.36)	0.18	1.05 (1.00, 1.10)	0.04	1.13 (1.01, 1.27)	0.04

*FC* Fold changes for an increase in PM of 10 μg/m^3^ (short- and medium-term) and 2 μg/m^3^ (long-term). Adjusted for age, BMI, smoking status, educational level, and time of blood sampling, temperature, WBC count, and percentage of neutrophils. Mt.: mitochondrial

^a^3 outliers with relatively low *POLG* expression removed

^b^1 outlier with relatively high *LONP1* expression removed. Results including the outlier were similar (long-term PM_2.5_ p-val = 0.007). FDR-adjusted *p*-values <0.05^$$^ and <0.10^$^

### Mitochondrial DNA content

Mitochondrial DNA content was negatively associated with short-, medium-, and long-term PM_10_ and PM_2.5_ exposure in women (Table 7). For men, mtDNA content was negatively associated with short-term PM_10_ and PM_2.5_ exposure whilst medium-term PM_10_ and PM_2.5_ exposure revealed a trend towards significance. No significant associations were observed between mtDNA content and the expression of the 13 selected Human MitoCarta genes.

**Table 7:** Association between mtDNA content and PM exposure

Time window	Men (*n* = 67)		Women (*n* = 83)	
	FC (95% CI)	*P*-val	FC (95% CI)	*P*-val
Short-term PM_10_	0.80 (0.67, 0.96)	0.02	0.82 (0.69, 0.97)	0.02
Short-term PM_2.5_	0.82 (0.69, 0.98)	0.03	0.83 (0.7, 0.98)	0.04
Medium-term PM_10_	0.77 (0.57, 1.04)	0.09	0.74 (0.55, 0.99)	0.05
Medium-term PM_2.5_	0.75 (0.55, 1.03)	0.08	0.73 (0.53, 1.01)	0.06
Long-term PM_10_	0.95 (0.87, 1.04)	0.26	0.9 (0.83, 0.97)	0.007
Long-term PM_2.5_	0.88 (0.72, 1.08)	0.22	0.76 (0.64, 0.91)	0.004

*FC* Fold changes for an increase in PM of 10 μg/m^3^ (short- and medium-term) and 2 μg/m^3^ (long-term). Adjusted for age, BMI, smoking status, educational level, and time of blood sampling, temperature and WBC count and percentage of neutrophils

## Discussion

The current study identified several mitochondrial-related genes and pathways significantly associated with fine particle exposure at different exposure time windows: short- (one week before the blood sampling) and medium-term (one month before the blood sampling) PM_10_ exposure. For women, PM exposure affected, among others, pathways contributing to mitochondrial genome maintenance (short-term), electron transport chains (short-, medium-term) and mitochondrial translation (short- and medium-term). For men, the TCA cycle was positively associated with medium-term PM_10_ exposure. Furthermore, we were able to validate a selection of mitochondrial-linked genes in an independent study population.

Transcriptome-wide long-term (two-year averages) results of the discovery cohort were described in a previous paper identifying potential gene expression biomarkers of PM exposure [14]. In line with the results for medium-term exposure, the electron transport chain was significantly associated with long-term PM_10_ and PM_2.5_ exposure in women.

For women, we selected three genes (*COX7C, UQCRH,* and *NDUFA13*), associated with medium-term PM_10_ exposure in the discovery cohort, encoding proteins contributing to the electron transport chain complexes and four genes (*POLG*, *STOML2, DNAJA3,* and *LONP1*) significantly associated with short-term PM_10_ exposure in the discovery cohort, of which their corresponding proteins play a role in mitochondrial genome maintenance. For all genes, the direction of association, by PM exposure during the significant time window of the discovery cohort, were replicated in the validation cohort by qPCR. We validated *POLG*, *LONP1*, *COX7C* and *UQCRH* in relation to long-term PM exposure and *DNAJA3* and *LONP1*, for medium-term exposure. For men, none of the selected TCA contributing genes could be validated. However, consistent with the results for women, *UQCRH,* and *NDUFA13* were upregulated and *LONP1* was down-regulated by long-term PM exposure in the validation cohort. Possibly, we could validate most genes only for long-term exposure because this exposure estimate is independent of season of blood sampling, which partly differs between the two study cohorts. For the validation cohort more pronounced effects were observed for PM_2.5_ compared to PM_10_, however, for each time window the correlation coefficient between PM_2.5_ and PM_10_ was >0.85.

In accordance with our study results, Hoffmann and colleagues observed significantly increased ETC protein levels (Complex II, III, V) in the human bronchial epithelial cell line BEAS-2B in response to cigarette smoke exposure for 6 months [30]. The upregulated expression of ETC genes (Fig. 1), as observed in the current study, and ETC proteins due to environmental toxicant exposure may indicate increased energy demand required to eliminate damage to cellular components.

**Fig. 3:**
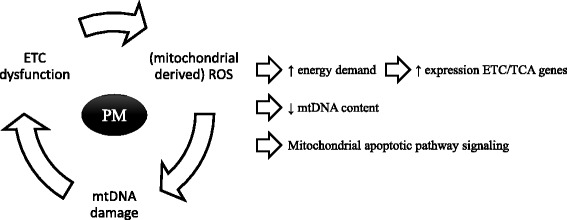
Schematic representation of the hypothesised pathway by which PM exposure alters mitochondrial functioning and genome maintenance. Within the mitochondria, PM can interact with the electron transport chain inducing increased levels of ROS production. ROS can damage mtDNA leading to further mitochondrial dysfunctioning and ROS production. To repair or eliminate damaged cellular components, the electron transport genes are upregulated to provide the required energy. Elimination of damaged mtDNA and perturbation of mtDNA replication results in a reduction of mtDNA content. Eventually, accumulation of mitochondrial damage can lead to mitochondrial apoptotic signalling. TCA: Tri Carbonic Acid cycle; ETC: electron transport chain; mtDNA: mitochondrial DNA

To further explore mitochondrial responses to PM exposure, we investigated mtDNA content in the validation cohort. In line with the results at the gene expression level (downregulation of genes important for mitochondrial genome replication such as *POLG*, *POLG2*, and *POLRMT*), mtDNA content, measured in the validation cohort, was decreased among both women and men exposed to relatively high PM levels for short-, medium- and long-term. To date, several studies reported a deregulation of mtDNA content in response to environmental factors [3, 25, 31–34]. However, the direction of effect is not consistent over these studies. Differences in exposure levels and population characteristics often make it difficult to compare study findings. The findings of the current study are in line with previous evidence reporting the ability of ultrafine particles to induce oxidative stress and mitochondrial damage [35], and the selective elimination of damaged mtDNA in order to help maintain mtDNA integrity [4]. Cline hypothesised that poly aromatic hydrocarbons, toxic components of PM, can block mtDNA polymerase and topoisomerase activity and in turn reduce mtDNA replication [3]. In this study, we observe a reduction at the level of gene expression of *POLG*, *POLG2* and *POLRMT* which may further explain the decreased mtDNA content in individuals exposed to relatively high PM exposure. Presumably, decreased mtDNA content and mitochondrial damage stimulate transcription factors regulating the expression of electron transport genes in order to provide the required energy to eliminate cellular damage. Mitochondrial dysfunction can augment ROS production which may in turn activate the mitochondrial apoptotic pathway. The altered expression of pro- and anti-apoptotic genes (such as gene members of the BCL-2 family and caspases) in response to short-term PM_10_ exposure in women and of members of the PI3K/AKT (busulfan) pathway, which delivers an anti-apoptotic signal, in men further supports the theory that PM-induced formation of ROS can influence mitochondrial function and regulate cell fate [36]. Fig. 3 shows a schematic overview of the potential effect of PM on mitochondria.

Overall, differences were observed between the response to PM exposure in men and women. However, for both sexes interacting pathways are altered by PM_2.5_ exposure; the mitochondrial apoptotic pathway is tightly regulated by several factors such as Bcl-2 family members, altered in women, and the PI3K/AKT (busulfan) pathway modulated in men [37, 38]. Moreover, the TCA cycle, deregulated in men, donates high-energy molecules to the ETC of which genes were differently expressed by PM exposure in women of both cohorts and in men of the validation cohort. In the validation cohort, the effects of air pollution on mtDNA content and expression levels of respiratory electron chain genes and genes contributing to the mitochondrial genome maintenance were more pronounced in women compared to men. Possibly, men are more effectively protected against environmental toxicants and ROS as implied by previous studies [39, 40]. Both studies reported more oxidative damage in female smokers compared to male smokers [39, 40]. In accordance, we observed in men exposed to relatively high PM levels, augmented expression levels of some aldehyde dehydrogenases (*ALDH7A1*↑, *ALDH1B1*↑), which convert reactive aldehydes (produced by oxidation of unsaturated fatty acids by ROS) to less toxic products, whilst in women *ALDH7A1* was down-regulated.

A strength of our study is that we validated genes in an independent validation cohort by means of qPCR. Moreover, in addition to gene expression, we analyzed mtDNA content in regard to PM exposure in the validation cohort. Our study has some limitations. First, observational studies do not allow to establish causality. Second, PM_2.5_ estimates were only available for the validation cohort. Third, the large number of tests in combination with the observational study design reduces the power of the transcriptome-wide study. However, this was addressed through focused analyses on MitoCarta genes and mitochondrial pathways using the ORA approach.

## Conclusions

Peripheral blood mtDNA content and expression of several genes related to mitochondrial genome maintenance, apoptosis and energy production were altered by PM exposure in a population of healthy middle-aged men and women, potentially reflecting mitochondrial and cellular damage. Future studies at different omics level may further clarify the effect of air pollution on mitochondria functioning and biogenesis.

## Additional file

Additional file 1: Figure S1. Volcano plots for short-term exposure (A: women, B: men) and for medium-term PM_10_ exposure (C: women, D: men). (DOCX 492 kb)

## Abbreviations

AT: Apparent temperature
BMI: Body mass index
CI: Confidence interval
*C*_*p*_: Threshold cycle
ETC: Electron transport chain
FDR: False discovery rate
FLEHS: Flemish environment and health survey
GO: Gene Ontology
HPRT: Hypoxanthine phosphoribosyltransferase 1
IFDM: Immission frequency distribution model
IPO8: Importine 8
IRCEL: Belgian interregional environment agency
mtDNA: Mitochondrial DNA
MT-ND1: Mitochondrially encoded NADH dehydrogenase 1
ORA: Overrepresentation analysis
PM: Particulate matter
PM_10_: Particulate matter with a diameter < 10 μm
PM_2.5_: Particulate matter with a diameter < 2.5 μm
qPCR: Quantitative real-time polymerase chain reaction
qRT-PCR: Real-time quantitative PCR
ROS: Reactive oxygen species
TCA: Tri carbonic acid
WBC: White blood cell
WHO: World Health Organization
YWHAZ: Tyrosine 3-monooxygenase/tryptophan 5-monooxygenase activation protein, zeta

## References

[1] Aon MA, Cortassa S, O'Rourke B (2010). Redox-optimized ROS balance: a unifying hypothesis. Biochim Biophys Acta.

[2] Lee HC, Wei YH (2005). Mitochondrial biogenesis and mitochondrial DNA maintenance of mammalian cells under oxidative stress. Int J Biochem Cell Biol.

[3] Cline SD (1819). Mitochondrial DNA damage and its consequences for mitochondrial gene expression. Biochim Biophys Acta.

[4] Liu P, Demple B (2010). DNA repair in mammalian mitochondria: much more than we thought?. Environ Mol Mutagen.

[5] Paradies G, Petrosillo G, Pistolese M, Ruggiero FM (2002). Reactive oxygen species affect mitochondrial electron transport complex I activity through oxidative cardiolipin damage. Gene.

[6] Li X, Fang P, Mai J, Choi ET, Wang H, Yang XF (2013). Targeting mitochondrial reactive oxygen species as novel therapy for inflammatory diseases and cancers. J Hematol Oncol.

[7] Wallace DC (2010). Colloquium paper: bioenergetics, the origins of complexity, and the ascent of man. Proc Natl Acad Sci U S A.

[8] Sahin E, Colla S, Liesa M, Moslehi J, Muller FL, Guo M (2011). Telomere dysfunction induces metabolic and mitochondrial compromise. Nature.

[9] Lin MT, Beal MF (2006). Mitochondrial dysfunction and oxidative stress in neurodegenerative diseases. Nature.

[10] Boland ML, Chourasia AH, Macleod KF (2013). Mitochondrial dysfunction in cancer. Front Oncol.

[11] Pagliarini DJ, Calvo SE, Chang B, Sheth SA, Vafai SB, Ong SE (2008). A mitochondrial protein compendium elucidates complex I disease biology. Cell.

[12] Calvo SE, Clauser KR, Mootha VK (2016). MitoCarta2.0: an updated inventory of mammalian mitochondrial proteins. Nucleic Acids Res.

[13] van Leeuwen DM, Gottschalk RW, Schoeters G, van Larebeke NA, Nelen V, Baeyens WF (2008). Transcriptome analysis in peripheral blood of humans exposed to environmental carcinogens: a promising new biomarker in environmental health studies. Environ Health Perspect.

[14] Vrijens K, Winckelmans E, Tsamou M, Baeyens W, De Boever P, Jennen D et al. Sex-specific Associations between Particulate Matter Exposure and Gene Expression in Independent Discovery and Validation Cohorts of Middle-aged Men and Women. Environ Health Perspect. In Press. DOI: 10.1289/EHP370. 10.1289/EHP370 PMC5381989 27740511

[15] Janssen S, Dumont G, Fierens F, Mensink C (2008). Spatial interpolation of air pollution measurements using CORINE land cover data. Atmos Environ.

[16] Maiheu B, Veldeman B, Viaene P, De Ridder K, Lauwaet D, Smeets N et al. Identifying the best available large-scale concentration maps for air quality in Belgium. 2012. Available from: http://www.milieurapport.be/Upload/main/0_onderzoeksrapporten/2013/Eindrapport_Concentratiekaarten_29_01_2013_TW.pdf.

[17] Lefebvre W, Degrawe B, Beckx C, Vanhulsel M, Kochan B, Bellemans T (2013). Presentation and evaluation of an integrated model chain to respond to traffic- and health-related policy questions. Environ Model Softw.

[18] Olesen H (1995). The model validation exercise at Mol: overview of results. Int J Environ Pollut.

[19] Maes G, Cosemans G, Kretzschmar J, Janssen L, Van Tongerloo J (1995). Comparison of six Gaussian dispersion models used for regulatory purposes in different countries of the EU. Int J Environ Pollut.

[20] Cosemans G, Kretzschmar J, Janssen L, Maes G (1995). The third workshops environmental impact assessment model intercomparison exercise. Int J Environ Pollut.

[21] Mensink C, Maes G (1996). Comparative sensitivity study for operational short-range atmospheric dispersion models. Int J Environ Pollut.

[22] Cosemans G, Ruts R, Kretzschmar JG. Impact assessment with the Belgian dispersion model IFDM and the New Dutch National Model. Belgitrate: 7th Int Conf on Harmonisation within Atmospheric Dispersion Modelling for Regulatory Purposes; 2001:125–9.

[23] Steadman RG (1979). The assessment of sultriness. Part II: effects of wind, extra radiation and barometric pressure on apparent temperature. J Appl Meteorol.

[24] Kalkstein LS, Valimont KM. An Evaluation of Summer Discomfort in the United-States Using a Relative Climatological Index. Bulletin of the American Meteorological Society. 1986;67:842–8.

[25] Janssen BG, Munters E, Pieters N, Smeets K, Cox B, Cuypers A (2012). Placental mitochondrial DNA content and particulate air pollution during in utero life. Environ Health Perspect.

[26] Hellemans J, Mortier G, De Paepe A, Speleman F, Vandesompele J (2007). qBase relative quantification framework and software for management and automated analysis of real-time quantitative PCR data. Genome Biol.

[27] Faner R, Gonzalez N, Cruz T, Kalko SG, Agusti A (2014). Systemic inflammatory response to smoking in chronic obstructive pulmonary disease: evidence of a gender effect. PLoS One.

[28] Paul S, Amundson SA. Differential Effect of Active Smoking on Gene Expression in Male and Female Smokers. J Carcinog Mutagen. 2014;5:198. 10.4172/2157-2518.1000198 PMC4303254 25621181

[29] Kamburov A, Pentchev K, Galicka H, Wierling C, Lehrach H, Herwig R (2011). ConsensusPathDB: toward a more complete picture of cell biology. Nucleic Acids Res.

[30] Hoffmann RF, Zarrintan S, Brandenburg SM, Kol A, de Bruin HG, Jafari S (2013). Prolonged cigarette smoke exposure alters mitochondrial structure and function in airway epithelial cells. Respir Res.

[31] Bonner MR, Shen M, Liu CS, Divita M, He X, Lan Q (2009). Mitochondrial DNA content and lung cancer risk in Xuan Wei. China Lung Cancer.

[32] Hou L, Zhu ZZ, Zhang X, Nordio F, Bonzini M, Schwartz J (2010). Airborne particulate matter and mitochondrial damage: a cross-sectional study. Environ Health.

[33] Zhong J, Cayir A, Trevisi L, Sanchez-Guerra M, Lin X, Peng C (2016). Traffic-related air pollution, blood pressure, and adaptive response of mitochondrial abundance. Circulation.

[34] Pieters N, Koppen G, Smeets K, Napierska D, Plusquin M, De Prins S (2013). Decreased mitochondrial DNA content in association with exposure to polycyclic aromatic hydrocarbons in house dust during wintertime: from a population enquiry to cell culture. PLoS One.

[35] Li N, Sioutas C, Cho A, Schmitz D, Misra C, Sempf J (2003). Ultrafine particulate pollutants induce oxidative stress and mitochondrial damage. Environ Health Perspect.

[36] Dagher Z, Garcon G, Billet S, Gosset P, Ledoux F, Courcot D (2006). Activation of different pathways of apoptosis by air pollution particulate matter (PM2.5) in human epithelial lung cells (L132) in culture. Toxicology.

[37] Engelman JA (2009). Targeting PI3K signalling in cancer: opportunities, challenges and limitations. Nat Rev Cancer.

[38] Fulda S (2013). Modulation of mitochondrial apoptosis by PI3K inhibitors. Mitochondrion.

[39] Hakim IA, Harris R, Garland L, Cordova CA, Mikhael DM, Sherry Chow HH (2012). Gender difference in systemic oxidative stress and antioxidant capacity in current and former heavy smokers. Cancer Epidemiol Biomark Prev.

[40] Mooney LA, Perera FP, Van Bennekum AM, Blaner WS, Karkoszka J, Covey L (2001). Gender differences in autoantibodies to oxidative DNA base damage in cigarette smokers. Cancer Epidemiol Biomark Prev.

