# Supplementary material for: Transcriptome-wide analyses indicate mitochondrial responses to particulate air pollution exposure
Source: Environ Health. 2017 Aug 18;16:87. doi: 10.1186/s12940-017-0292-7 (PMC5563023; doi:10.1186/s12940-017-0292-7)
Supplement: Additional file 1: Figure S1. — Volcano plots for short-term exposure (A: women, B: men) and for medium-term PM10 exposure (C: women, D: men). (DOCX 492 kb) [file 12940_2017_292_MOESM1_ESM.docx]

**Supplementary Material**

**Transcriptome-wide analyses indicate mitochondrial responses to particulate air pollution exposure**

Ellen Winckelmans, Tim S Nawrot, Maria Tsamou, Elly Den Hond, Willy Baeyens, Jos Kleinjans, Wouter Lefebvre, Nicolas Van Larebeke, Martien Peusens, Michelle Plusquin, Hans Reynders, Greet Schoeters, Charlotte Vanpoucke, Theo M. de Kok, Karen Vrijens


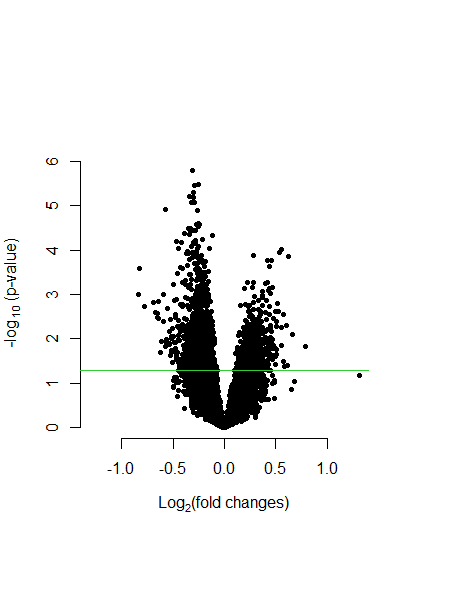

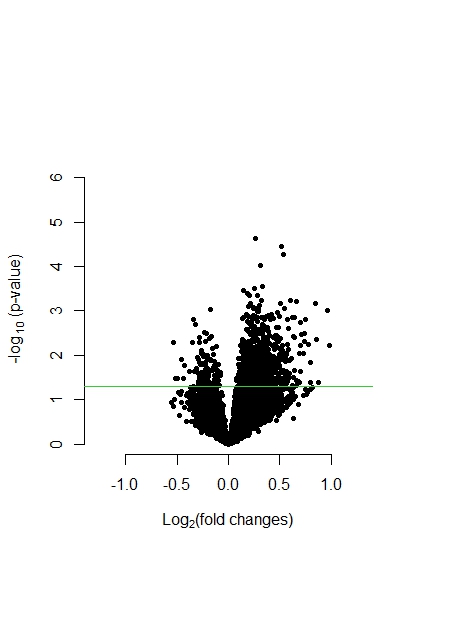

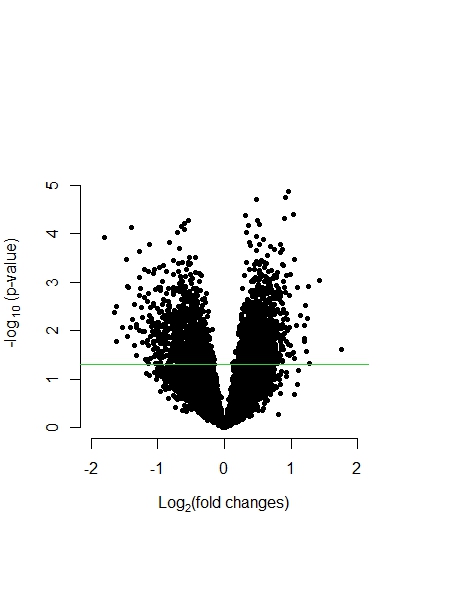

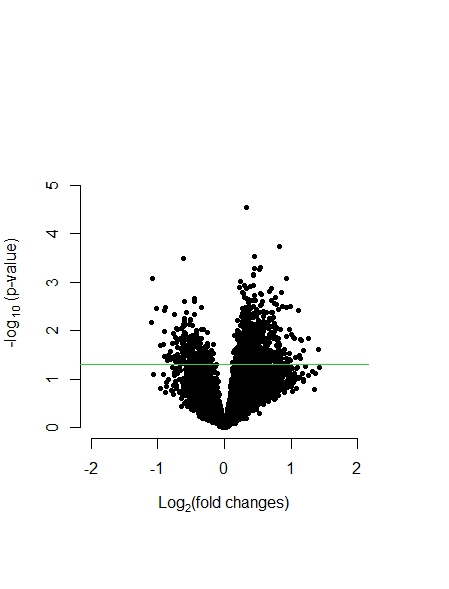


A.

B.

C.

D.

Short-term

Short-term

Medium-term

Medium-term

Women

Men

Log_2_ (Fold change)

Log_2_ (Fold change)

Log_2_ (Fold change)

Log_2_ (Fold change)

-log_10_ (p-value)

-log_10_ (p-value)

-log_10_ (p-value)

-log_10_ (p-value)

Figure S1. Volcano plots for short-term exposure (A: women, B: men) and for medium-term PM_10_ exposure (C: women, D: men). Log_2_ fold changes are given for an increase in PM_10_ exposure of 10 µg/m^3^.
